# Inertial Sensor-Based Lower Limb Joint Kinematics: A Methodological Systematic Review

**DOI:** 10.3390/s20030673

**Published:** 2020-01-26

**Authors:** Ive Weygers, Manon Kok, Marco Konings, Hans Hallez, Henri De Vroey, Kurt Claeys

**Affiliations:** 1 KU Leuven Campus Bruges, Department of Rehabilitation Sciences, 8200 Bruges, Belgium; marco.konings@kuleuven.be (M.K.); henri.devroey@kuleuven.be (H.D.V.); kurt.claeys@kuleuven.be (K.C.); 2 TU Delft, Department of Mechanical and Materials Engineering, 2628 CD Delft, The Netherlands; M.Kok-1@tudelft.nl; 3 KU Leuven Campus Bruges, Department of Computer Science, Mechatronics Research Group, 8200 Bruges, Belgium; hans.hallez@kuleuven.be

**Keywords:** inertial measurement unit, lower quadrant, movement analysis, outside laboratory, sensor fusion

## Abstract

The use of inertial measurement units (IMUs) has gained popularity for the estimation of lower limb kinematics. However, implementations in clinical practice are still lacking. The aim of this review is twofold—to evaluate the methodological requirements for IMU-based joint kinematic estimation to be applicable in a clinical setting, and to suggest future research directions. Studies within the PubMed, Web Of Science and EMBASE databases were screened for eligibility, based on the following inclusion criteria: (1) studies must include a methodological description of how kinematic variables were obtained for the lower limb, (2) kinematic data must have been acquired by means of IMUs, (3) studies must have validated the implemented method against a golden standard reference system. Information on study characteristics, signal processing characteristics and study results was assessed and discussed. This review shows that methods for lower limb joint kinematics are inherently application dependent. Sensor restrictions are generally compensated with biomechanically inspired assumptions and prior information. Awareness of the possible adaptations in the IMU-based kinematic estimates by incorporating such prior information and assumptions is necessary, before drawing clinical decisions. Future research should focus on alternative validation methods, subject-specific IMU-based biomechanical joint models and disturbed movement patterns in real-world settings.

## 1. Introduction

Evaluating kinematical characteristics is crucial for a correct clinical understanding of complex functional movements such as gait [1], a forward lunge and other tasks requiring optimal motor control [2]. Studying kinematics can help in the assessment of the patients’ functionality and progression in their rehabilitation period. Different lab-based methods are currently available for researchers to obtain kinematical parameters.

A 3D optical motion capture system is currently the gold standard and the most commonly used technique to study lower limb movement [3]. However, optical motion capture systems require a rather expensive set-up of infrared cameras that track reflective markers attached to the body of a subject. This type of movement analysis is therefore only applicable in a dedicated laboratory environment and thus is restricted in physical space. Furthermore, the accuracy in the obtained joint kinematics directly relates to a correct placement of markers [4,5] and soft tissue artifacts [3].

To overcome these restrictions, the use of wearable devices to monitor human movements has been studied extensively [6,7]. Recent reviews concerning kinematic analysis with inertial measurement units (IMUs) are typically conducted either by engineering experts [7,8,9] or by clinicians [10,11,12], who focus on technical aspects or clinical relevance. Previously conducted reviews highlighted the growing interest for inertial sensors in clinical practice [13]. Benson et al. [1] reported the need for gait analysis over longer time periods, with larger number of participants, in natural environments. O’Reilly et al. [14] pointed towards the use of machine learning techniques for lower limb exercise detection and classification with IMUs. Moreover, Picerno [12] presented a history of methodologies for IMU-based joint kinematic estimation of the past 25 years in gait analysis.

However, when applying IMU-based joint kinematics to specific applications in a clinical setting, a good understanding of the methodological requirements is still lacking. The aim of this review is twofold—to evaluate the methodological requirements for IMU-based, lower limb joint kinematic estimation to be applicable in a clinical setting, and to suggest future research directions.

## 2. Methods

### 2.1. Eligibility Criteria

This review focused on peer-reviewed articles and conference papers published in English which included a description of the methodology used to obtain kinematic variables of the lower limb by means of IMUs. To be included in the review, a validation against a reference system e.g., an optical motion capture system, should be reported. Also, studies of which the methodology was extended with measurement modalities other than these available within IMUs, i.e., a pressure sensor, were included. A journal paper was preferred over a conference paper when similar content was covered.

Review papers and book chapters were excluded. Studies related to upper quadrant movements were not considered for this review. Studies of which the methodology was not applicable for outside-laboratory usage were left out. Articles lacking a description of a reproducible algorithm to obtain joint kinematics were also excluded.

### 2.2. Search Strategy and Study Selection

**Figure 1:**
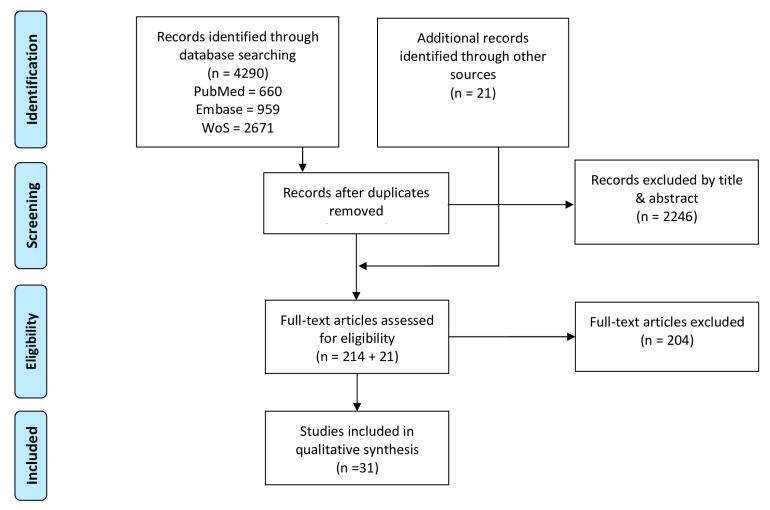
Preferred Reporting Items for Systematic review and Meta-Analyses (PRISMA) flowchart of the search strategy and study selection procedure, adapted from [15].

The available literature was searched in a systematic way through a personalized four step PRISMA method (Preferred Reporting Items for Systematic review and Meta-Analyses). These four steps are summarized in Figure 1. A systematic literature search in three databases (PubMed, Web of Science, EMBASE) covering a broad range of both medical and engineering studies was conducted. The literature was updated on a monthly basis until the end of September 2019. The search strategy and combinations of keywords for the different databases are described in Appendix A. After the removal of duplicates, the remaining records were screened on title and abstract and assessed for eligibility. Additionally, references of included articles were screened to ensure inclusion of all relevant studies.

### 2.3. Data Extraction

Data from all selected articles were extracted and are structured in Table 1, Table 2 and Table 3. The following information was extracted: (1) study characteristics (Table 1) covering participant information, the activity assessed, activity duration and the joints of interest with their investigated degrees of freedom (DoF). Additionally, sensor placement and the used sensor modalities contribute to the study characteristics, where sensor modalities refers to the raw sensor data that are used in the algorithm. Later in the article, IMU will be used for inertial measurement units which can measure specific force, angular velocity and sometimes magnetic field strength. (2) Signal processing characteristics (Table 2) describing the process to obtain meaningful kinematic measures from measured sensor data. This process is divided into pre-processing, the additional assumptions that have been made and the signal processing techniques that are used to extract the kinematic measures. The pre-processing is related both to sensor calibration as well as to filtering of the raw sensor signals. Later in the article, priors and assumptions will be defined as follows—prior information relates to information acquired by ether auxiliary apparatus or estimated by IMU measurements, while assumptions relate to a certain hypothesis made. Signal processing techniques describe how to cope with sensor to segment calibration, orientation initialization, sensor fusion and drift compensation. (3) The study results (Table 3) summarize the accuracy of the proposed methods against a reference system.

## 3. Results

The results of our systematic literature search initially identified 4290 articles of which thirty-one articles were ultimately included. Extracted information of included studies on study characteristics (Table 1), signal processing characteristics (Table 2) and study results (Table 3) are covered in respectively Section 3.1, Section 3.2 and Section 3.3.

### 3.1. Study Characteristics

The study characteristics summarized in Table 1 cover participant information, protocol of the conducted study and sensor set-up. Only four out of the thirty-one included studies involved more than ten participants [16,17,18,19]. Furthermore, a predominantly healthy population was recruited, except for identified populations with ankle osteoarthritis [17], transfemoral amputation [20], incomplete spinal cord injury [21], children with cerebral palsy (CP) [19] and stroke patients [22].

Among the thirty-one studies included in this review, gait was the most commonly evaluated activity, which was assessed either on a treadmill [16,17,23,24] or a walkway [20,21,22,25,26,27,28,29,30,31,32,33,34,35,36,37,38]. Functional movements (i.e., sit-to-stand, squat) are the next most common activities [18,19,32,39,40,41]. Furthermore, seven studies focused on more dynamic types of locomotion such as common daily activities [24] and sports, including ski racing [42], running [38,43,44], standing long jump [45] and cycling [46]. Only nine studies reported kinematic analysis beyond 30s of recording [17,21,22,24,29,42,43,44,46].

Most articles did not study the full 3D kinematics of the entire lower quadrant. Sagittal knee joint movements were most extensively studied [21,23,36,40,43,44]. Furthermore, three studies extended such a hinge joint model to three DoF knee joint movements [25,26,29]. Two studies solely assessed the hip joint in two DoF [28] and three DoF [46]. Two studies solely focused on the ankle complex in three DoF [17,32]. Studies that incorporated multiple joints, also predominantly restrict movement analysis to the sagittal plane. To this end, most commonly assessed was the combination of the full sagittal lower limb (hip, knee, ankle) [24,30,31,34,38,41,45]. Second most common combination was the assessment of the sagittal knee and ankle joint movement [16,19,20,27].

Nearly all the studies made use of sensors placed on adjacent segments surrounding the joint of interest. Four studies opted for a more distal placement on the segments [18,22,35,37]. Only three out of thirty-one studies were able to provide a reproducible description of sensor placement based on anatomical landmarks [19,38,42]. Most of the studies only used three-axes gyroscopes and three-axes accelerometers [17,18,19,20,22,25,26,30,31,33,34,37,38,39,42,44]. Six studies additionally incorporated a magnetometer [24,29,32,43,45,46]. Moreover, four studies complemented IMU measurements with other modalities, such as a pressure sensor [21,24,36,45].

### 3.2. Signal Processing Characteristics

Signal processing characteristics (Table 2) were split up into three components. The first component covered pre-processing and the use of prior information and assumptions. Sensor readings are typically corrupted by measurement noise and bias. These noise and bias affected measures require a compensation in the form of pre-processing steps. The incorporation of prior knowledge and assumptions will further ease the calculation of kinematics. A second component summarized methods to combine pre-processed measurements from different sensor modalities with priors and assumptions, called information fusion. The third component described compensation mechanisms to account for integration drift, to initialize joint angles, and to cope with a natural misplacement of a sensor with respect to the boney landmarks.

#### 3.2.1. Pre-Processing, Prior Information and Assumptions

Eight studies compensated for gyroscope bias [18,21,22,32,33,34,37,42]. Seven studies described a method for accelerometer calibration [16,18,22,32,33,38,42]. More extensive calibration procedures for inertial measurement units [47,48] were subsequently used in [18,22,32,38,42]. Raw inertial sensor measurements were often filtered. Most commonly, a Butterworth filter [19,21,32,34,39,46] was used. The filter orderwas chosen between 2 and 4. Cut-off frequencies differed between 2Hz and 20Hz, with the exception of [42], where information above 100 Hz was neglected.

The analysis of human motion yields biomechanically related prior information such as joint range of motion (RoM) [18,39,41], segment lengths [18,24,33,34,37,38,40,41,45] and spatiotemporal gait parameters [21,37]. Additionally, the indirect way of measuring kinematics with IMUs placed on the body segments required prior knowledge about the sensor position with respect to the joint center [23,24,29,40,46], sensor to segment alignment [17,29,34,37,40,44,46] and an initial sensor orientation or kinematic estimate [39,44]. Furthermore, three studies described a method to learn a fully subject-specific model [30,31,43].

Besides prior knowledge, specific assumptions were also exploited to ease the estimation of joint kinematics. Both a single description of joint acceleration in a common reference frame (up to a time-variant rotation matrix) and segment rigidity were often used as assumptions [20,23,28,29,33,35,36,38,42]. Furthermore, human motion was assumed to coincide with kinematic chain conventions [49,50] that describe how connected segments behave [18,22,37,39]. Seven studies assumed periodicity in the motion [16,17,22,30,38,39,41,45]. Moreover, three studies assumed symmetry between the right and left side of the subject [35,41,45].

#### 3.2.2. Information Fusion

A set of common approaches for information fusion were identified in the thirty-one studies considered in this review. Strap-down integration was used [17,19,25,26,32,34,35,42,45] and characterized by Sabatini et al. [51] as “The attitude of the rigid body and its non-gravitational accelerations are sensed by gyroscopes and accelerometers strapped to the body.” Two studies followed a complementary filtering approach which acts in the frequency domain, to simultaneously filter high-frequency noise on the accelerometer inclination estimates and low-frequency noise on the integrated angular velocity relative orientation estimates [20,46]. Estimating sensor orientations is an inherently nonlinear problem. Five studies made use of nonlinear filtering techniques, such as an extended Kalman filter, to optimally fuse sensor measurements [18,22,24,37,44] or nonlinear numerical optimization [29,38,39] which makes use of all data samples to obtain a kinematic estimate.

Different from conventional information fusion methods, other methods were proposed for the application of joint kinematic estimation. For example, three studies used regression techniques to map raw sensor data to known kinematic parameters [30,31,43]. Furthermore in [33,40], motions were modeled as a moving pendulum to implicitly handle the fusion. Inverse kinematics were aided with accelerometer displacement estimates in [29,41,45]. Furthermore, Dejnabadi et al. [23] described joint angles at the joint center, based on the assumption of a common joint acceleration for one physical point. In contrast with information fusion, the use of only one sensor modality was exploited in six studies to overcome the need for fusion in accelerometer-only [16,27,28,36,40] and gyroscope-only methods [21].

#### 3.2.3. Drift Compensation, Initialization and Calibration

Integration drift originates as an artifact by integration of noise- and bias-affected gyroscope measures. In this article, global and relative drift are defined as drift with respect to a global or an adjacent segment reference frame, respectively. Drift in the vertical plane was compensated by tilt estimates from accelerometer readings, which are dominated by gravity during characteristic samples [17,19,20,24,25,26,27,32,34,35,42]. Further drift compensation in the horizontal plane was achieved by utilizing magnetometer readings [32,46]. Two studies reported a resetting approach for azimuth angles in a cycle-by-cycle manner during mid-stance in gait [17,45]. Regardless of the movement plane, relative drift between segments is compensated by the incorporation of priors and assumptions. For example, imposing segments to be connected at all times [29] by imposing joint DoF boundaries [44] or RoM constraints [18]. Also, the incorporation of time periods when segments were connected to the ground [18,21,24,37,38] were used to reset to the known absolute inclinations at given time instances. Furthermore, distinguishing gravity from linear acceleration by modeling motion as a pendulum was used to prevent drift [46]. One study reported on the reduction of drift solely by means of filtering raw sensor measurements in a pre-processing step [34]. Furthermore, four studies reported the use of a periodicity [22,38,39,41] assumption to compensate for drift.

The information fusion techniques from Section 3.2.2 require the kinematics at the first instance to be initialized. A reasonable initialization can be obtained using prior knowledge. Six studies described the need for a static upright pose to obtain an initial orientation [16,25,26,27,29,42,45]. Three studies extended the latter by means of two different static poses [23,33,34]. Furthermore, magnetometer data were used to initialize the azimuth angle [29]. The initial difference between segments around the vertical was identified by means of ab/adduction movement [25,26]. Furthermore, two studies utilized auxiliary apparatus such as photogrammetric devices to initialize the sensor orientation [39,44].

To overcome misalignment between sensor and segments, different approaches were identified. Two studies aligned the sensor and segment with active [26] or passive [32] sagittal and frontal shank movements. One study extended calibration movements to application-dependent movements [42,52]. Five studies exploited prior knowledge to overcome sensor to segment misalignments [29,34,37,40,44,46]. In contrast to the aforementioned calibration methods, two studies assumed a perfect alignment between sensor and anatomical axis [21,23].

### 3.3. Study Evaluation and Results

Study validity with respect to a reference system is reported in Table 3. For the reader’s convenience, the least accurate results were considered in all studies that report multiple results for different activities or methodological parameters. When available, the results for healthy and pathological subjects were reported separately. Accuracy by means of root mean squared errors, mean absolute error or correlation coefficients of the estimated angles was reported with respect to a reference system. References systems other than an optical motion capture system consisted of an ultra-sound system [23], magnetic tracking device [25,26], flexible goniometer [16] and high definition cameras [42]. Two studies showed a disagreement in degrees of freedom in (Table 1) and the validated degrees of freedom [37,42]. Inconsistent accuracy results were reported for all joints of the lower limb. Sagittal plane motions for all studies were reported with mean RMSE values ranging between 1.3–11.22 degrees while frontal and transversal RMSE values ranged between 1–6.7 and 1.4–6.5 degrees, respectively.

## 4. Discussion

This review systematically evaluated the methodological requirements for IMU-based lower limb joint kinematic estimation. Human motion analysis with inertial sensors has the potential to increase understanding in movement patterns in trusted well-known environments [10]. However, from an engineering point of view, it is an ambitious goal that is currently the subject of research [1,12]. A general inconsistency in accuracy of the study results (Table 3) indicates that the signal processing characteristics summarized in Section 3.2 (Table 2) highly depend on the application (Table 1) of interest.

In summary, lower limb kinematic estimation from inertial sensors requires a well-defined application and study characteristics. The study characteristics define which sensor modalities will be measured and processed to compensate for the following sensor restrictions: (1) due to their microelectromechanical architecture, raw sensor measurements are prone to noise and non-zero biases; (2) an integration step of measurements is typically necessary to obtain joint kinematics, resulting in drifting estimates of sensor orientations and joint kinematics; (3) inertial sensors are usually not aligned with the bone, which implies that misalignment with respect to anatomical coordinate frames needs to be identified; (4) initial sensor orientations need to be determined.

In order to overcome these sensor restrictions, all of the included articles were required to rely in some way on application-specific prior information and assumptions. However, by including this additional information in the methodology, the resulting kinematic estimates need to be interpreted carefully, taking into consideration a number of factors, before drawing any clinical decision.

First, the biomechanical system yields usable prior information, but this information can be violated in practical applications. For example, assumptions on the range of motion can restrict the kinematic solution to be within a given interval of normal physical ability. However, RoM boundaries are not generalizable across patient populations who might be hypermobile or hypomobile, exceeding or not reaching normal RoM respectively. Also, segment lengths are relevant priors that can be obtained, as described by Crabolu et al. [56]. Multiple studies make use of an estimated vector that describes the position of the center of the joint in the sensor’s coordinate frame [57]. Such joint center position vectors implicitly assume that segments are rigid and connected at one common fixed point. However, possible small joint-translational movements and soft tissue artifacts will violate this model [58,59]. In reality, soft tissue artifacts are present when patients move [60]. Frick et al. [58,59] recently proposed a method that identifies the time variations of a joint center position vector due to soft skin movement, but lacks a proper validation. Ideally, prior knowledge is estimated from sensor measurements [20,46,56] rather than measured in a movement laboratory or obtained from anthropometric tables.

Second, assuming periodicity in motion dates back to Morris et al. [61], to solve for integration drift by making the beginning and ending of a gait trial equal [62]. Still, a more relaxed assumption on periodicity, instead of resetting, is more convenient [17]. Two studies compensate for integration drift in azimuth angles on a cycle-by-cycle manner during mid-stance in gait [17,45]. Nevertheless, the latter is not a measure of absolute heading and might lead to accumulating errors on the foot progression angle [63]. Along the same lines, symmetry assumptions can help to allow for reducing the number of sensors on the body [35,41], but might over-constrain the system. For example, Bonnet et al. [41] analyzed the execution of a squat movement with a symmetry assumption on the legs. The method intends to only use one sensor, placed at the lower back. However, by applying a symmetry assumption, frontal hip, knee, and pelvis motion are not assessed, while still very relevant in such transitional movements [64]. Multiple studies utilized a zero-acceleration assumption at the contact point of the foot with the ground and therefore expected one foot to be on to the ground on a regular basis. Note that such an assumption might become invalid when applied to movements that lack a regular mid-stance phase such as running or other arbitrary movements.

Moreover, calibration movements are typically required to obtain a misalignment matrix between the sensor and anatomical reference frames. However, predefined calibration movement with a fully extended leg can be difficult within certain patient populations or during post-op periods [25,42]. As a result, the precision of the calibration depends on the accuracy with which the subject or instructor performs the calibration movements. A trend towards calibration-free methods with arbitrary placement of sensors and the avoidance of calibration movements is visible [20,65,66].

The incorporation of additional information such as assumptions and prior information can easily be done in an optimization-based smoothing approach for applications that demand high accuracy [29,39]. Solving such problem in a smoothing way, implicitly uses all available data [29,38] instead of a one-way filtering approach with only samples of the past. On the other hand, biofeedback applications ask for computationally less expensive fusion methods that can provide real-time estimates such as complementary filters [54,67,68].

## 5. Future Research

This review highlights the application dependency and inherent connection of methodological characteristics for lower limb IMU-based kinematic estimation. Assumptions and prior information are typically used to compensate for sensor limitations and to enhance the quality of kinematic estimates. Because of this, IMU-based kinematic estimates have to be interpreted carefully, before drawing any clinical decision. We identified a number of directions and pieces of advice for future research for the estimation of clinically relevant lower limb kinematics.

### 5.1. Reporting Joint Kinematics

For the clinical interpretability of the joint kinematics, the general reporting standards from the International Society of Biomechanics (ISB) [69,70] need to be followed. Only seven out of thirty-one of the included articles mentioned these standards. Joint kinematics are described as the movement of a distal segment with respect to its proximal segment, following a joint coordinate system [71]. The following movements are clinically relevant: (1) flexion/extension movements in the sagittal plane that occurs around the proximal segment-fixed frontal axis; (2) internal/external movements around the body-fixed longitudinal axis of the distal joint describing movements in the transversal plane; (3) abduction and adduction movements around the floating axis perpendicular to the two previously mentioned axes, describing frontal plane movement.

### 5.2. Biomechanical Joint Modeling

Gait predominantly occurs in the sagittal plane, and therefore the knee is often modeled as a hinge joint. A hinge joint axis can be estimated from IMU readings [20,66] to compensate for the misalignment between sensor and bone, which allows for an arbitrary placement of the sensing units. However, smaller joint movement in frontal and transversal planes also occurs and plays a critical role in for example ligament injuries [72,73,74]. Investigation in more complex and even subject-specific tibiofemoral joint models with inertial sensors, may provide highly valuable inside in these secondary joint kinematics for outside laboratory applications [75].

Furthermore, a recent trend is visible towards the inclusion of multiple joints and segments, rather than estimating kinematics for separate joints [29,38,42]. When multiple sensors are exploited, common information can be used to improve kinematic estimates. In this case, an appropriate joint model needs to be chosen for each individual joint [76].

### 5.3. Validation with Respect to a Golden Standard Reference

Optical motion capture systems are the most commonly used technique to study lower limb movement. They are therefore also most often used as a reference to evaluate IMU-based joint kinematic estimation methods. However, due to manual marker placement errors [77] and soft tissue artifacts [5,78], a conventional 3D gait analysis system will introduce biases that are predominantly present in smaller frontal and transversal movements. These secondary joint kinematics yield valuable insight into ligament loading and ACL injury [72]. Stagni et al. [5] concluded that flexion/extension at the knee by means of optical external markers can be considered acceptably reliable. However, internal/external rotations and ab/adduction at the knee are critically affected by soft-tissue artifacts. In order to validate internal/external rotations and ab/adduction kinematic estimated by means of IMUs, alternative validation methods (i.e., biplanar radiographic imaging systems [79,80]) that might be superior in tracking underlying bone movements need to be examined.

### 5.4. Measurement Duration and Environment

Whilst IMUs are proposed for long term observations, there are still few studies tackling measurements beyond 30 s. With respect to long, in-the-wild studies, measurement duration must be increased [81,82,83]. Resolving this problem could potentially bring the use of IMUs closer to applications in which subjects can be monitored for hours or days, with bursts of activity in-between long in-activity periods [81,82,83]. To meet this requirement, a clear trend is visible towards magnetometer-free methods, only acquiring accelerometer and gyroscope readings. The authors of this review believe that this idea is important, specifically for outside-lab applicability (i.e., hospital environment, sports field), without the need for assumptions on magnetic field homogeneity.

### 5.5. Disturbed Movement Patterns

Most of the published work recruited young, healthy participants. However, in clinics, most attention must go to the investigation of different patient populations with disturbed daily functional movement such as gait, sit-to-stand, stand-to-sit or climbing stairs [64]. One of the crucial aspects here is the inability of the patient (e.g., patients with neuromotor disorders or people with severe limb disorders) to perform pre-defined calibration movements, often necessary for the evaluation of functional movements with IMUs. The eligibility criteria in Section 2.1 demand a reproducible description of the algorithm. This might have resulted in studies that lack extensive validation on disturbed movement patterns, which is often done in a later phase, e.g., [84]. Investigating disturbed movement patterns and calibration-free methods to cope with sensor-to-segment misalignment in different patient populations will be an important avenue of research.

## 6. Conclusions

This review systematically evaluated the methodological requirements for IMU-based lower limb joint kinematic estimation. *Where are we now?* Despite the ongoing research regarding the computation of joint kinematics by means of IMUs, there still appear to be difficulties which prevent their use in daily clinical practice. It is reasonable to assume that the complexity in obtaining meaningful kinematic measures from noisy and biased measured sensor data and sensor restrictions regarding integration drift, sensor-to-segment alignment and initial sensor orientation explain these study restrictions. *What can already be measured with sufficient accuracy?* Most often, biomechanically inspired assumptions and prior information are used to compensate for sensor limitations. Both clinicians and engineers have to be aware of the possible adaptations in the IMU-based kinematic estimates by incorporating such prior information and assumptions, before drawing clinical decisions. *What needs to be tackled with high priority?* Investigating the appropriate validation methods that might be superior in tracking underlying bone movement and can overcome the restrictions of optical motion capture systems as a reference. *What might yield novel results?* Subject-specific IMU-based biomechanical joint models applied to populations with disturbed movement patterns in real-world settings. Combined efforts of engineers and clinical experts can result in application- and patient-specific implementations that will be valuable to clinicians.

## Figures and Tables

**Table 1:** Study characteristics.

Ref.	Participants	Protocol			Sensor Set-Up		
	Number and Sex, Type, Age	Activity	Duration	DoF Joint of Interest	Placement		Modalities
[23]	5M 3F, healthy,	Gait (Treadmill)	30s	1DoF knee	thigh, shank		1D GYR, 2D ACC
	range: 44-70 mean = 58.7	(2–4 km/h)					
[25]	10M, healthy,	Gait	10s	3DoF knee	thigh, shank		3D GYR, 3D ACC
	range: 23-40 mean = 29						
[26]	8M, healthy,	Gait	5s *	3DoF knee	thigh, shank		3D GYR, 3D ACC
	range 19–28 mean = 26						
[16]	27, healthy,	Gait (Treadmill) (0,15 m/s–2 m/s)	6s *	1DoF knee,	thigh, shank, foot		2× 2D ACC
	mean = 26 SD = 1,5			1DoF ankle			
[27]	10, healthy,	Gait	3s *	1DoF knee,	thigh, shank, foot		2× 2D ACC
	mean = 31 SD = 4	(slow, normal, fast)		1DoF ankle			
[28]	6M 2F, healthy,	Gait	10s *	2DoF hip	close to the hip, thigh		2× 3D ACC,
	mean = 25 SD = 3	(slow, normal, fast)					1× 3D ACC
[42]	6, European cup level alpine ski racers, ND	Alpine ski racing	90s *	3DoF hip,	sternum, sacrum, thigh (lateral mid-distance between the knee and hip joint center), shank (tibial plateau)		3D GYR, 3D ACC
				3DoF knee			
[29]	ND	Gait	35s *	3DoF knee	pelvis, thigh, shank, foot		3D GYR, 3D ACC, 3D MAG
[30]	8, healthy, ND	Gait	ND	1DoF hip,	shank, foot		3D GYR, 3D ACC
		(slow, normal, fast)		1DoF knee,			
				1DoF ankle			
[31]	8, healthy, ND	Gait	12s *	1DoF hip,	shank, foot		3D GYR, 3D ACC
		(self-selected speed)		1DoF knee, 1DoF ankle			
[43]	8M, healthy experienced runners, mean = 25.1 SD = 5.2	Running (Treadmill)	180s	1DoF knee	pelvis, shank		3D GYR, 3D ACC, 3D MAG
		(10 km/h–14 km/h)					
[32]	2M, healthy, range: 23–25	Leg exercises, gait	8s *	3DoF ankle	3DoF ankle		3D GYR, 3D ACC, 3D MAG
[40]	1M, ND, 29	Squat	11s *	1DoF knee	thigh, shank		1D ACC
[20]	1, transfemoral amputee, 40	Gait	6s *	1DoF knee, 1DoF ankle	thigh, shank, foot		3D GYR, 3D ACC
[33]	3M, healthy, range: 23–28	Gait	10s *	2DoF hip, 1DoF knee	thigh, shank		3D GYR, 3D ACC
[34]	5M, healthy, range: 22–27	Gait	ND	1DoF hip, 1DoF knee, 1DoF ankle	pelvis, thigh, shank, foot		3D GYR, 3D ACC
[24]	5M, healthy, mean = 27.6 SD = 3.4	Gait (Treadmill),	15min	1DoF hip, 1DoF knee, 1DoF ankle	lower back, thigh, shank, foot		3D GYR, 3D ACC, 3D MAG, pressure insoles
		Stair walking					
[35]	6, healthy, > = 18	Gait	ND	1DoF hip, 1DoF knee	center of lumbar, thigh (most distal), shank (most distal)		1D ACC, 1D GYR
		(Cadence range: 60-120 step/min)					
[36]	1, healthy, ND	Gait (self-selected speed)	2s *	1DoF knee	thigh, shank		2 × 3D ACC, PSECR insole
[17]	7F 3M/3F 9M, healthy/unilateral ankle osteoarthritis, mean = 60 SD = 15/mean = 61 SD = 13	Gait (Treadmill)	5min	3DoF shank-hindfoot, 3DoF hindfoot-forefoot, 3DoF shank-forefoot, 3DoF forefoot-toes	shank, hindfoot, forefoot, toes		3D GYR, 3D ACC
		(2 km/h–5 km/h)					
[45]	1M, healthy, 23	Standing	5s *	1DoF hip, 1DoF knee, 1DoF ankle	chest, right thigh, right shank		3D GYR, 3D ACC, 3D MAG, pressure insole
		long jump					
[18]	12M 8F, healthy, mean = 23	Functional rehabilitation exercises	ND	3DoF (stationary base), 2DoF knee, 1DoF	hip (height of the anterior superior iliac spine), thigh (near the knee), calf (near the ankle)		3D GYR, 3D ACC
[41]	5M 3F, healthy, mean = 32.5 SD = 9.9		Squat	ND	1DoF hip, 1DoF knee, 1DoF ankle	lower back	1D GYR, 2D ACC
[39]	9M 1F, healthy, mean = 25 SD = 3		Functional exercise	ND	3DoF hip, 1DoF knee	shank	1D GYR, 2D ACC
[37]	5, healthy, range: 19–25		Gait	ND	3DoF hip, 1DoF knee, 1DoF ankle	waist, hip, knee	3D GYR, 3D ACC
[46]	1, ND, ND		Cycling	5min	3DoF hip	pelvis, thigh	3D GYR, 3D ACC, 3D MAG
[21]	2, 1 healthy and 1 incomplete SCI subject, ND		Gait	50s *	1DoF knee	thigh, shank	1D GYR, FSR
[19]	5 healthy/28 CP, 3M 2F/17M 3M 2F/17M 11F, healthy/CP, mean = 26 SD = 2.0/(18 subjects mean 7.5 sd = 3.1 and 10 subjects mean 5.5 sd 3.5)		Leg movements in supine position	30s *	1DoF knee, 1DoF ankle	thigh, shank, foot	3D GYR, 3D ACC
[22]	3 and 2, healthy and stroke patients, ND and rang: 67:77		Gait	80s	3DoF hip, 1DoF knee	pelvis, thigh, shank	3D GYR, 3D ACC
[44]	5M 3F, healthy, mean = 30 SD = 6		Gait, running	5min	1DoF knee	thigh, shank	3D GYR, 3D ACC
[38]	10M, healthy, mean = 27.1 sd = 2.6		Gait, running	ND	1DoF hip, 1DoF knee, 1DoF ankle	lower back, lateral thigh, lateral shank, and upper midfoot.	3D GYR, 3D ACC

Abbreviations: ND = not described; N/A = not applicable; M = Men; F = Female; SD = standard deviation; GYR = gyroscope; ACC = accelerometer; MAG = magnetometer; D = Dimensions (number of sensitive axes); DoF = degree of freedom; * = activity duration not explicitly mentioned, based on time axis of plots incorporating sampling frequencies; PSECR = pressure sensitive electric conductive rubber; SCI = incomplete spinal cord injured; FSR = force sensitive resistors.

**Table 2:** Signal processing characteristics.

Ref.	Pre-Processing		Additional Information		Signal Processing		
	Sensor Calibration	Filter, Type, Order, Cut-Off, Input Data	Prior Knowledge	Assumptions	Initialization and Sensor-Segment Alignment	Information Fusion	Drift Compensation
[23]	ND	Savitzky-Golay, LP, 3, ND, GYR ACC	Position sensor w.r.t. joint center (Photography)	Same acceleration in joint center, Sensor-to-segment mounting assumptions	(INI) Static: 5s (knees extended) and 5s (knees flexed) (S-S) assumptions	Virtual sensors in joint center	N/A
[25]	ND	ND	ND	ND	(INI) Static: 10s stand still, Functional: hip AA.	Strap-down integration [53]	ACC gravity compensation, during characteristic samples
[26]	ND	ND	ND	ND	(INI) Static: stand still Functional: hip AA, (S-S) Passive shank movements in frontal and sagittal plane	Strap-down integration [53]	ACC gravity compensation, during characteristic samples
[16]	ACC	ND	ND	Tangential and centripetal acceleration from redundant ACC set-up, Periodicity	(INI) Static: 2s stand still before and after a trial	N/A	BP filtering
[27]	ND	ND	Position sensor w.r.t. joint center	Tangential and centripetal acceleration from redundant ACC set-up	(INI) Static: 2s stand still before and after a trial	N/A	ACC gravity compensation, during characteristic samples, Nonlinear optimization
[28]	ND	ND, LP, ND, 20Hz, ACC	Position sensor w.r.t. joint center	Tangential and centripetal acceleration from redundant ACC set-up, Same acceleration in joint center	ND	N/A	N/A
[42]	GYR, ACC [47]	ND, LP, ND, 100, GYR ACC	ND	Same acceleration in joint center	(INI) Static: stand still (S-S)[52]	Strap-down integration [53]	ACC gravity compensation, during characteristic samples, Multi-sensor drift correction
[29]	GYR	ND	Manual measuring: Orientation sensor on body segment, Position sensor w.r.t. joint center	Segments are connected to each other at all time and Sensors can move slightly w.r.t. body segment	(INI) Static: stand still pose (ACC and MAG) (S-S) prior information	Constraint optimization, Strap-down integration	Exploit assumptions
[30]	ND	ND	Subject specific trained model	Periodicity	ND	Regression: GRNN	N/A
[31]	ND	ND	Subject specific trained model	ND	ND	Regression: GRNN	N/A
[43]	ND	(No inertial data was filtered)	Subject specific trained model	ND	ND	Regression: ANN, two-layer (250 and 100 neurons)	N/A
[32]	GYR, ACC [47]	Butterworth, LP, 2, 5Hz, ACC GYR	ND	Assume same discrepancies in magnetic field interference for both segments	(S-S) Functional: two leg movements.	Strap-down integration	ACC gravity compensation, during characteristic samples, MAG readings
[40]	ND	ND	Stereo-photogrammetric: Segment lengths, Orientation sensor on body segment, Position sensor w.r.t. joint center	Feet are supposed rigidly connected to the ground, Pendulum motion	(S-S) prior information	N/A	N/A
[20]	ND	ND	ND	Same acceleration in joint center	N/A	Complementary filter	Exploit assumptions
[33]	GYR, ACC	Moving average, LP, ND, 15 point at 100Hz, ACC GYR	Segment lengths	Same acceleration in joint center, Pendulum motion thigh around the hip, constant velocity in walking speed	(S-S) Static: two poses (standing upright and sitting flat with outstretched legs)	Pendulum model	N/A
[34]	GYR	Butterworth, LP, 4, 12Hz, GYR	Segment lengths, Orientation sensor on body segment (sagittal image)	ND	(INI) Static: two poses (standing upright and sitting flat with outstretched legs) (S-S) prior knowledge	Strap-down integration	ACC gravity compensation, during characteristic samples
[24]	ND	ND	Optical reference: Segment lengths, Position sensor w.r.t. joint center	One foot on the ground at all time with zero acceleration at contact point.	ND	Recursive EKF	ACC gravity compensation, during characteristic samples, Exploit assumptions
[35]	ND	ND	Position sensor w.r.t. joint center	Symmetry, Drift linearly accumulates during integration, ACC measured most distal at a segment equals the joint center acceleration, Same acceleration in joint center	ND	Strap-down integration	ACC gravity compensation, during characteristic samples, Modeling and correcting drift as linear accumulating
[36]	ND	ND, BP, ND, function of PSECR skin motion frequency content, ACC	Position sensor w.r.t. joint center, total pressure, CoP between sensor and skin	Same acceleration in joint center	ND	N/A	N/A
[17]	ND	ND	Orientation sensor on body segment (Optical motion capture system)	Periodicity	(S-S) prior information	Strap-down integration	ACC gravity compensation, during characteristic samples, Azimuth zeroing cycle-by- cycle.
[45]	ND	ND	Segment lengths	Symmetry, Periodicity	(INI) Static: stand still pose.	Strap-down integration, displacement from ACC double integration	Exploit assumption Symmetry
[18]	GYR, ACC [47]	ND	Segment lengths (Optical motion capture system), RoM, (anthropometric data)	DH convention	ND	EKF	Exploit assumptions & prior information
[41]	ND	ND	Segment lengths, RoM	Periodicity, perfect sagittal symmetry	ND	Inverse kinematics, displacement from ACC double integration	Exploit assumptions
[39]	ND	Butterworth, LP, ND, 2Hz, squared GYR	RoM, Segment lengths (manually measured), Initial joint angle (measured by goniometer)	Periodicity, DH convention	(INI) prior information	Constraint optimization	Exploit assumptions
[37]	GYR	ND	Segment lengths, Orientation sensor on body segment, (Manual annotation) Spatio-temporal data.	One foot on the ground at all time, kinematic chain model [49]	(S-S) prior information	EKF	Exploit assumptions
[46]	ND	Butterworth, LP, 4, 15Hz, ACC	Position sensor w.r.t. joint center, Orientation sensor on body segment	Pendulum motion of thigh segment around the hip CoR	(S-S) prior information	Adapted complimentary filter [54]	Exploit assumptions, MAG readings
[21]	GYR	Butterworth, LP, 4, 4Hz, GYR	Spatio-temporal data (FSR measurements)	Sensor-to-segment mounting assumptions	(S-S) assumptions	N/A	HP filtering kinematic estimates, resetting during mid-stance in gait cycle.
[19]	ND	Butterworth, LP, 2, 10Hz, GYR ACC	ND	Movements outside sagittal plane occur only at full extension	ND	Strap-down integration	ACC measuring gravity during characteristic samples. Correction for movements outside of sagittal plane.
[22]	GYR, ACC [48]	ND, LP, ND, 10Hz, GYR ACC	ND	Periodicity, kinematic chain model [49]	ND	Adapted EKF	Exploit assumption Periodicity
[44]	ND	ND	Sensor orientation difference adjacent segments, Initial sensor orientation (Optical motion capture system)	ND	(CAL and S-S) prior information	EKF	Exploit Joint DoF constraint.
[38]	GYR, ACC [47]	ND	Segment masses, measured segment lengths, center of mass locations, moments of inertia [55]	Periodicity, constrain translational joint movement	(S-S) ND (I-I) initial guess	Constraint optimization	Exploit assumptions

Abbreviations: ND = not described; N/A = not applicable; GYR = gyroscope; ACC = accelerometer; AA = Abduction Adduction; LP = Low Pass; BP = Band Pass; S-S = Sensor to Segment alignment; INI = initial orientation; MAG = magnetometer; GRNN = General Regression Neural Network; ANN = Artificial Neural Network; EKF = Extended Kalman filter; PSECR = pressure sensitive electric conductive rubber; CoP = Center of Pressure; DH = Denavit-Hartenberg; RoM = Range of Motion; DoF = Degrees of Freedom.

**Table 3:** Study results.

Ref.	Reference	Accuracy			
	Method	Joint [Measure, Unit]	Sagittal	Frontal	Transversal
[23]	Ultra-sound based motion measurement	knee [RMSE, deg]	1.3		
[25]	Magnetic tracking device	knee [RMSE mean (SD), deg]	1.5 (0.4) *	1.7 (0.5) *	1.6 (0.5) *
[26]	Magnetic tracking device	knee [RMSE mean (SD), deg]	1.3 (0.5) *	2.0 (0.6) *	2.0 (0.9) *
[16]	Flexible goniometer	knee [RMSE median, deg]	5		
		ankle [RMSE median, deg]	3.5		
[27]	Optical motion capture system	knee [RMSE median, deg]	3		
		ankle [RMSE median, deg]	2.8		
[28]	Optical motion capture system	hip [RMSE, deg]	4.1	4.9	
[42]	High definition camera’s	hip [RMSE, deg]	6.0 (1.5) *	ND	ND
		knee [RMSE, deg]	4.8 (1.7) *	ND	ND
[29]	Optical motion capture system	knee	graph only	graph only	graph only
[30]	Optical motion capture system	hip	*^,^*^,^*		
		knee	*^,^*^,^*		
		ankle	*^,^*^,^*		
[31]	Optical motion capture system	hip [correlation]	(0.7-0.89) *^,^*^,^*		
		knee [correlation]	(0.7-0.89) *^,^*^,^*		
		ankle [correlation]	(0.7-0.89) *^,^*^,^*		
[43]	Optical motion capture system	knee [mean RMSE, deg]	<5 *^,^*^,^*		
[32]	Optical motion capture system	ankle [RMSE, deg]	[0,1] *^,^*	[1–2.5] *^,^*	[2.5–4.5] *^,^*
[40]	Mechanical pendulum setup and Optical motion capture system	knee [RMSE mean (SD), deg]	1.01 (0.11)		
[20]	Optical motion capture system	knee [RMSE healthy|prosthesis mean (SD), deg]	3.30 (1.20)|0.71 (0.19)		
		Ankle [RMSE healthy|prosthesis, deg]	1.62 (0.57)|0.81 (0.16)		
[33]	Optical motion capture system	hip [RMSE, deg]	8.72	4.96	
		knee [RMSE, deg]	6.79		
[34]	Optical motion capture system	hip [RMSE, deg]	10.14		
		knee [RMSE, deg]	7.88		
		ankle [RMSE, deg]	9.75		
[24]	Optical motion capture system	hip (median over all described conditions, deg)	<5		
		knee (median over all described conditions, deg)	<5		
		ankle (median over all described conditions, deg)	<5		
[35]	Optical motion capture system	hip [RMSE mean (SD), deg]	5.24 (0.27)		
		knee [RMSE mean (SD), deg]	11.22 (1.09)		
[36]	Optical motion capture system	knee	graph only		
[17]	Optical motion capture system	shank-hindfoot, hindfoot-forefoot, shank-forefoot, forefoot-toes [RMSE, deg]	[1.4; 2]	[1.4; 2]	[1.4; 2]
[45]	Optical motion capture system	hip [RMSE, deg]	3.92		
		knee [RMSE, deg]	7.87		
		ankle [RMSE, deg]	3.22		
[18]	Optical motion capture system	hip [RMSE mean, deg]	4.3	6.5	6.5
		knee [RMSE mean, deg]	4.3	6.5	
		ankle [RMSE mean, deg]	4.3		
[41]	Optical motion capture system	hip [RMSE mean (SD), deg]	3.1 (0.9)		
		knee [RMSE mean (SD), deg]	2.0 (1.0)		
		ankle [RMSE mean (SD), deg]	3.2 (1.0)		
[39]	Optical motion capture system	hip [RMSE mean (SD), deg]	3.6 (2.4)	2.4 (1.0)	2.7 (1.4)
		knee [RMSE mean (SD), deg]	4.0 (3.1)		
[37]	Optical motion capture system	hip	ND	ND	ND
		knee [RMSE mean (SD), deg]	6.20 (1.48)		
		ankle	ND		
[46]	Optical motion capture system	hip [MAE, deg]	0.8	6.7	2.2
[21]	Optical motion capture system	knee [RMSE, deg]	6.42		
[19]	Optical motion capture system	knee [RMSE, deg]	<3		
		ankle [RMSE, deg]	<3		
[22]	Optical motion capture system	hip [RMSE, deg]	2.4	2.4	2.4
		knee [RMSE, deg]	2.4		
[44]	Optical motion capture system	knee [RMSE, deg]			
[38]	Optical motion capture system	hip [RMSE mean (SD), deg]	8.7		
		knee [RMSE mean (SD), deg]	5.3		
		ankle [RMSE mean (SD), deg]	4.6		

Abbreviations: ND = not described; deg = degree; SD = standard deviation; *(precision) after removing an offset; *,* interpreted from box-plot *,*,* Inter and intra-subject dependent.

## Appendix A

Combination of keywords:

*Web Of Science*

TS = (inertial measurement unit OR imu OR accelerometer OR magnetometer OR gyroscope OR inertial sensor*) AND TS = (kinematic* OR joint angle*OR joint angle velocity OR joint angle acceleration)

*PubMed*

‘((kinematic*[tiab] OR joint angle*[tiab] OR “joint angle velocity”[tiab])) AND (“inertial measurement unit”[tiab] OR imu[tiab] OR accelerometer[tiab] OR magnetometer[tiab] OR gyroscope[tiab] OR inertial sensor*[tiab])’

*Embase*

(‘kinematics’/exp OR ‘kinematic*’:ti,ab OR ‘joint angle*’:ti,ab OR ‘joint angle velocity’:ti,ab OR ‘joint angle acceleration’:ti,ab OR ‘joint angle’/exp) AND (‘inertial measurement unit’/exp OR ‘inertial measurement unit’:ti,ab OR ‘imu’:ti,ab OR ‘accelerometer’:ti,ab OR ‘magnetometer’:ti,ab OR ‘gyroscope’:ti,ab OR ‘inertial sensor*’:ti,ab OR ‘accelerometer’/exp OR ‘magnetometer’/exp OR ‘gyroscope’/exp)
